# Long-term health-related and economic consequences of short-term outcomes in evaluation of perinatal interventions

**DOI:** 10.1186/1471-2393-10-42

**Published:** 2010-08-10

**Authors:** Margreet J Teune, Aleid G van Wassenaer, Ben Willem J Mol, Brent C Opmeer

**Affiliations:** 1 Academic Medical Center Amsterdam, The Netherlands

## Abstract

**Background:**

Many perinatal interventions are performed to improve long-term neonatal outcome. To evaluate the long-term effect of a perinatal intervention follow-up of the child after discharge from the hospital is necessary because serious sequelae from perinatal complications frequently manifest themselves only after several years. However, long-term follow-up is time-consuming, is not in the awareness of obstetricians, is expensive and falls outside the funding-period of most obstetric studies. Consequently, short-term outcomes are often reported instead of the primary long-term end-point. With this project, we will assess the current state of affairs concerning follow-up after obstetric RCTs and we will develop multivariable prediction models for different long-term health outcomes. Furthermore, we would like to encourage other researchers participating in follow-up studies after large obstetric trials (> 350 women) to inform us about their studies so that we can include their follow-up study in our systematic review. We would invite these researchers also to join our effort and to collaborate with us on the external validation of our prediction models.

**Methods/Design:**

A systematic review of neonatal follow-up after obstetric studies will be performed. All reviews of the Cochrane Pregnancy and Childbirth group will be assessed for reviews on interventions that aimed to improve neonatal outcome. Reviews on interventions primary looking at other aspects than neonatal outcome such as labour progress will also be included when these interventions can change the outcome of the neonate on the short or long-term. Our review will be limited to RCTs with more than 350 women. Information that will be extracted from these RCTs will address whether, how and for how long follow-up has been performed. However, in many cases long-term follow-up of the infants will not be feasible. An alternative solution to limited follow-up could be to develop prediction models to estimate long-term health outcomes of the newborn based on specific perinatal outcomes and other covariates. For the development of multivariable prediction models for several health outcomes, we will use data available from a Dutch cohort study of preterm (< 32 weeks) and/or small for gestational age infants (< 1500 g). These infants were born in The Netherlands in 1983 and followed until they reached the age of 19.

**Discussion:**

The systematic review will provide insight in the extent and methods used for follow-up assessments after obstetric RCTs in the past. The prediction models can be used by future studies to extrapolate short-term outcomes to a long-term horizon or to indicate for which neonates long-term follow-up is required, as their outcomes (either absence or presence of sequelae) cannot be adequately predicted from short-term outcomes and clinical background characteristics.

## Background

Many perinatal interventions are performed to improve long-term neonatal outcome. To evaluate the long-term effect of a perinatal intervention follow-up of the child after discharge from the hospital is necessary because serious sequelae from perinatal complications frequently manifest themselves only after several years [1]. However, long-term follow-up is time-consuming, is not in the awareness of obstetricians, is expensive and falls outside the funding-period of most obstetric studies. Consequently, short-term outcomes are often reported instead of the primary long-term end-point.

The relevance of long-term outcomes is illustrated by the following two Cochrane reviews. In the first Cochrane review the implications of antibiotics for preterm rupture of membranes (erythromycin and/or amoxicillin-clavulanate) were evaluated. However, none of the 22 studies included in this review reported on long-term outcomes, but these data supported the routine use of antibiotics in premature rupture of membranes [2]. Recently, the results of the 7-year follow-up of one of these included studies were published. The prescription of antibiotics for women with preterm rupture of the membranes seems to have little effect on the health of children at 7 years of age, so the findings of decreased short-term morbidity from antibiotics have not translated in long-term benefits [3].

A second Cochrane review where women at risk for preterm labour with intact membranes were given prophylactic antibiotics (erythromycin and/or amoxicillin-clavulanate) identified 11 studies, without any study reporting long-term follow-up [4]. This review failed to demonstrate any overall benefit from prophylactic antibiotic treatment for preterm labour with intact membranes. Also recently, the long-term results of one of these included studies were published. The use of erythromycin by women at risk for preterm labour and with intact membranes was associated with an increase in functional impairment among their children at 7 years of age. The risk of cerebral palsy was increased by either antibiotic, although the overall risk of this condition was low [5]. These results support the advice that antibiotics are not recommended in spontaneous preterm labour with intact membranes and that the description of antibiotics can even be harmful in these circumstances.

If long-term follow-up of the infants is not feasible, an alternative is to model the long-term consequences based on short-term neonatal outcomes. This could be realised by developing prediction models, in which the association between short-term and long-term outcomes is determined statistically, and adjusted for relevant covariates. Models are a way of representing the complexity of the real world in a more simple and comprehensible form where true data are infeasible or impracticable to obtain. Modelling should be used as a last resource when RCTs or cohort studies do not provide all required information because they did not incorporate long enough follow-up [6-8]. Modelling has real strengths. It can be inexpensive, free of ethical concerns over treatment allocation and fast: a computer model can simulate in minutes a trial lasting years. Modelling has some less obvious benefits too, as the process of constructing the model promotes systematic thought and generated insights about the nature of its components and how they interact, which may help to identify areas in which empirical research is most needed, help generate new epidemiological or clinical hypotheses, and help produce novel ideas for useful interventions. Of course, modelling has its weak points. Failings in model theory or logic, inaccuracies in model parameters, or omission of key factors can all invalidate results [9].

Prediction models for long-term morbidity of infants could be used to extrapolate short-term outcomes or to indicate for which neonates long-term follow-up is required, as their outcomes (either absence or presence of sequelae) cannot be predicted from short-term outcomes and clinical background characteristics. The development of such models requires a longitudinal approach, in which data surrounding pregnancy, delivery and short-term outcomes are available, as well as follow-up data on various health related outcomes. The Dutch POPS cohort is one of the few birth cohorts in which this information has been systematically assessed. Data of infants born alive with a gestational age below 32 completed weeks and/or with a birth weight of 1500 gram were collected prospectively. A total of 1338 infants were included in this cohort, constituting 94% of the eligible infants born in 1983 in the Netherlands. Follow-up assessments were done at 2, 5 and 19 years of age [10-14]. This birth cohort could provide insight in the long-term consequences of perinatal outcomes and provides us with the necessary longitudinal data for developing prediction models for long-term health outcomes.

## Objectives of the project

In this project we aim to assess the current state of affairs on long-term follow-up after obstetric randomized controlled trials (RCTs). A systematic review will be performed to assess how often follow-up of infants after discharge of the hospital is carried out. Furthermore, we will develop multivariable prediction models for different long-term health outcomes which can be used by future studies to extrapolate their short-term outcomes to a long-term horizon. We would like to encourage other researchers participating in follow-up studies after large obstetric trials (> 350 women) to inform us about their studies so that we can include their follow-up study in our systematic review. Furthermore, we would invite these researchers to join our effort and to collaborate with us on the external validation of our prediction models.

## Methods/Design

### Systematic Review

We will search all reviews of the Cochrane Pregnancy and Childbirth group. Pubmed searches did not provide us with enough relevant studies on long-term outcomes, because of a lack of good MeSH terms. Therefore, we decided that we will search for these studies in the Cochrane library. We assumed that all important obstetric studies published will be included in one of the reviews of the Cochrane Pregnancy and Childbirth group. All reviews on perinatal interventions that aimed to improve neonatal outcome will be included. Reviews on interventions primary looking at other aspects than neonatal outcome such as labor progress will also be included when these interventions can change the outcome of the neonate on the short or long-term. Cochrane reviews of perinatal interventions that have no potentional health benefit for the neonate will be excluded (for example the Cochrane review "antibiotics regimens for endometritis after delivery"). All studies included in the relevant Cochrane reviews will be considered and only studies reporting RCTs with more than 350 women are included for subsequent analysis. Every included RCT will be screened for statements on long-term follow-up. RCTs without statements on long-term follow-up will be cross-checked in Web of Science to see if long-term effects are reported in subsequent publications. We assume that articles reporting on long-term effects of a specific perinatal intervention will refer to the original article reporting this RCT. Two independent reviewers will extract relevant information from these selected RCTs using a data collection sheet. The following characteristics will be extracted from included RCTs: general study characteristics (country, sample size, type of intervention, primary and secondary outcomes), whether long-term follow-up has been performed, and whether this follow-up was planned before the start of the RCT. If follow-up has been performed, we will also document duration of follow-up, follow-up rate and methods and instruments used during this follow-up. All information will be entered and analysed using SPSS 17.0 (SPSS Inc., Chicago, IL, USA).

### Multivariable prediction models

#### Study design

For the development of prediction models for several long-term health outcomes, we will use data available from a Dutch cohort study of preterm and/or small for gestational age infants (POPS study). In this cohort all live born infants were included, that were delivered in The Netherlands between January and December 1983, either before 32 completed weeks of gestation and/or with a birth weight of less than 1500 g. In total 1338 live born infants were included, 94% of all eligible infants. Follow-up assessments were done at 2, 5 and 19 years of age [10-14]. The follow-up rate after 2 years was 97%, after 5 years 96% and after 19 years 72%. This cohort provides valuable information on long-term consequences of perinatal outcomes.

#### Short-term outcomes

For each prediction model candidate predictors will be selected based on existing literature, combined with consulting experts in the field and the availability of these variables in the POPS cohort. Examples of these candidate predictors are social class, ethnicity, pregnancy induced hypertension, pre-eclampsia, diabetes gravidarum with diet or insulin, maternal smoking during the pregnancy, prolonged rupture of membranes, multiple pregnancy and caesarean section, gestational age at birth, birth weight, low Apgar scores, umbilical cord academia, respiratory distress syndrome (RDS), bronchopulmonary dysplasia (BPD), necrotizing enterocolitis (NEC), intraventricular haemorrhage (IVH), periventricular leukomalacia (PVL) and retinopathy of prematurity (ROP).

#### Long-term outcomes

Prediction models for the following long-term outcomes will be developed: respiratory and neurological morbidity, visual and hearing problems, intestinal morbidity and the costs associated with these medical conditions.

#### Statistical Analysis

We will develop multiple multivariable logistic regression models in which the association between short-term and long-term outcomes is determined statistically, and adjusted for relevant covariates. A power calculation is not possible at this stage because we have not yet defined our long-term outcomes and the potential predictors, and are unfamiliar with plausible distribution and association assumptions for the specific long-term outcomes.

We will use a multiple imputation approach to deal with missing values. Uncertainty about imputed values is reflected in differences between different imputed datasets, and incorporated in the estimated standard errors and associated p-values for each fitted model based on the pooled datasets.

For each prediction model a different selection of candidate predictors will be used. Univariable and multivariable regression analysis will be performed to estimate odds ratios (ORs), 95% confidence intervals (95% CI) and corresponding p-values for candidate predictors. As the use of too stringent p-values for variable selection is more deleterious for a model than including too many factors, all candidate predictors that showed a significance level of < 50% in univariable analyses will be entered in the multivariable logistic regression model [15]. Furthermore, we used a stepwise backward selection procedure, using a predefined significance level of > 20% for removing predictors from the models [8]. Discriminative capacity of the models will be evaluated by estimating the area under the receiver operating characteristic (ROC) curve. Calibration of the models will be assessed by comparing estimated probabilities with the observed proportion of respiratory morbidity. Goodness-of-fit will be tested formally with the Hosmer and Lemeshow test statistic. Data will be analysed using SPSS 17.0 (SPSS Inc., Chicago, IL, USA).

### Validation

Prediction models are known to be optimised for the specific dataset in which they have been derived. Internal and external validation is required to correct for this overfit bias, also known as optimism. Internal validation will be performed by uniform shrinkage or by bootstrapping of observations within the same dataset [8].

Furthermore, the models will be externally validated in other datasets available from ongoing or recently completed studies. For this validation, we will consider data from two Dutch studies (PETRA and TRUFFLE). In the PETRA-study, temporizing management with or without plasma volume expansion is compared for 216 women included between 1 April 2000 and 31 May 2003 [16]. Their infants will undergo neurological examination after 1, 5 and 10 years follow-up [17]. The TRUFFLE study currently investigates the best timing of delivery in preterm pregnancies complicated by intrauterine growth restriction, with neurological outcome measured at 2 years of age. In addition, we want to encourage researchers participating in other follow-up studies (longitudinal registries/birth cohorts) to contact us and to collaborate with our study to further explore the external validity and international generalizability of our prediction models for long-term outcomes. One important question to be addressed is whether these prediction models are also applicable to full-term neonates.

## Discussion

At the moment, it is not standard policy to do follow-up of the neonate after an obstetric RCT to evaluate long-term effects of a perinatal intervention. Main reasons for this limited follow-up are as follows; long-term follow-up is time-consuming, expensive and does not fall within the funding-period of most obstetric RCTs. It is also hypothesised that obstetricians may not be aware of important long-term consequences of their interventions. However, long-term follow-up is very important, as it can change the overall verdict regarding the optimal diagnostic or treatment strategy in a pregnant woman. The systematic review will provide insight in the extent and methods used for follow-up assessments after obstetric RCTs in the past. The different prediction models can be used by future studies to extrapolate their short-term outcomes to a long-term horizon or to indicate for which neonates long-term follow-up is required, as their outcomes (either absence or presence of sequelae) cannot be predicted from short term outcomes and clinical background characteristics.

Overall, the aim of this project is to increase international awareness of the importance of and to stimulate research on long-term follow-up after obstetric RCTs when evaluating perinatal interventions. We would like to encourage other researchers participating in large follow-up studies to join our effort and to collaborate with us on the external validation of our prediction models.

## Competing interests

The authors declare that they have no competing interests.

## Authors' contributions

BO is the principal investigator of the study described in this article. BO, BWM and AW developed the initial study protocol. BO, BWM and MT participated in the study design and coordination. MT wrote the first draft of the manuscript based on the study protocol. All other authors commented on this draft. All authors have read and approved the final manuscript.

## Authors' information

Margreet Teune is a medical doctor and studied Economics. Aleid van Wassenaer is a pediatrician. Ben Willem Mol is a gynecologist and clinical epidemiologist and is specialized in diagnostic and multicenter trial design. Brent Opmeer studied psychometrics and works as a clinical epidemiologist specialized in outcomes research.

## Pre-publication history

The pre-publication history for this paper can be accessed here:

http://www.biomedcentral.com/1471-2393/10/42/prepub
